# The Generation and Control of Harmful Products in Food Processing

**DOI:** 10.3390/foods12193679

**Published:** 2023-10-07

**Authors:** Xiaodong Lin, Biao Zhang, Maomao Zeng

**Affiliations:** 1 Zhuhai UM Science & Technology Research Institute, Zhuhai 519000, China; zumri.xdlin@um.edu.mo; 2 Zhejiang Provincial Key Laboratory of Biometrology and Inspection & Quarantine, College of Life Sciences, China Jiliang University, Hangzhou 310018, China; 3 State Key Laboratory of Food Science and Resources, Jiangnan University, Wuxi 214122, China

## 1. Introduction

Food processing is an integral part of the modern food industry aimed at improving the quality, taste, and preservation of food products. However, harmful compounds, such as heterocyclic amines, advanced glycation end products (AGEs), acrylamide ethyl carbamate, methylimidazole, and others, may be formed during these processing steps, posing potential health risks to consumers. In recent years, researchers have made significant progress in understanding and minimizing the harmful compounds produced during food processing, which has driven the development of effective strategies to detect, reduce, or eliminate these compounds in foods [1,2].

Scientists and researchers have been actively studying various aspects related to these harmful products, including developing detection methods, studying their formation mechanisms, exploring their presence in different food systems, and developing inhibition techniques to reduce their formation [3,4,5]. On the one hand, researchers have worked to develop and improve analytical techniques for the detection and quantification of these harmful compounds. Advanced methods, such as chromatography, mass spectrometry, immunoassay, polymerase chain reaction, nanomaterial-based biosensors, and point-of-care testing, have been applied to accurately identify and measure the levels of these compounds in food samples. These analytical data provide important information for risk assessment and regulation, contributing to ensure the safety of food products. On the other hand, understanding the mechanism of formation of these hazardous compounds is also one of the current research priorities. Certain factors, such as temperature, cooking method, time, and ingredient interactions, play an important role in the formation of these compounds. By studying these mechanisms, researchers can identify critical control points during food processing and develop strategies to minimize or prevent their formation, thereby reducing the potential risk to humans. Furthermore, researchers are investigating the occurrence and distribution of these harmful compounds in different food systems, which can help assess potential dietary exposures and prioritize areas of concern. It can also guide the development of targeted mitigation strategies, such as modification of processing parameters or introduction of new technologies, to reduce the levels of these compounds in specific food categories. The development of inhibitory technologies is also one of the main directions being explored in current research, investigating the use of antioxidants, enzymes, fermentation, heat treatments, and other innovative methods to inhibit or minimize the formation of these harmful products during food processing. These techniques are designed to protect the safety and quality of foods, while maintaining their sensory properties.

Currently, there is growing interest in the detection, mechanisms of production, presence, and inhibition of harmful compounds in food processing, reflecting growing concern for consumer health and the need to ensure the production of safe, high-quality food. Preventive measures, guidelines, and the development of new technologies can safeguard human health and improve the food processing industry as a whole.

## 2. Generation of Harmful Products in Food Processing

A number of harmful compounds may be produced during food processing that pose potential health risks, especially from prolonged exposure and intake of foods containing high levels of these substances. First, some of these hazardous products are considered to be potential carcinogens. For example, compounds such as heterocyclic amines, acrylamide, and methylimidazole have been associated with the development of certain cancers. Although the toxic and carcinogenic effects of these compounds are controversial in different studies, it is beneficial to reduce exposure to these compounds as a precautionary principle. Second, AGEs are harmful products that are formed during the reaction between sugars and proteins or fats. High levels of AGEs are strongly associated with the onset and progression of chronic diseases, such as diabetes, cardiovascular disease, and neurodegenerative diseases. These products produce oxidative stress and inflammatory responses to cells and tissues, and may damage blood vessel walls, tissues, and organ function. In addition, some hazardous products may exhibit irritant or toxic effects on specific organs or systems. Acrylamide ethyl carbamate may cause irritation to the respiratory system, skin, and eyes. 

It is worth noting that hazardous products from food processing are usually formed at high temperatures, over long periods of time, or under inappropriate operating conditions [6]. Therefore, proper food processing and cooking practices can help minimize the formation of harmful products. For instance, certain measures, such as choosing low-temperature cooking, shortening cooking time, and reducing the intake of fried and smoked foods, can help minimize the intake of harmful substances. Notably, the presence of harmful substances in food does not necessarily represent an immediate health hazard. The toxicity of a hazardous substance is related to a number of factors, such as intake, duration of exposure, individual sensitivities, etc. [7]. It is necessary to take these factors into account when assessing the safety of food and to follow national food safety standards and guidelines.

## 3. Control of Harmful Products in Food Processing

The control of harmful products in food processing is a critical aspect of ensuring food safety and quality. Hazardous product contaminants may present a risk to human health. Therefore, it is critical to adopt appropriate control measures and methods to reduce the risk of hazardous products. First of all, choosing high-quality raw materials is the first step in controlling hazardous products. Ensuring that these raw materials are fresh and free of contamination is an important factor in reducing the risk of harmful substances. Qualified supply chain management and proper screening of raw materials can help reduce sources of contamination. Subsequently, controlling the processing temperature and time are also important measures to manage hazardous products. Excessive temperatures and long processing times may lead to the formation of hazardous substances. Therefore, the temperature and time of processing should be strictly controlled to minimize the formation of harmful products. In addition, the rational use of food additives is also a key factor in controlling harmful products. Certain food additives may produce harmful substances under specific conditions. Therefore, food additives must be used in accordance with specified dosages and conditions of use to ensure safety and compliance. Good hygiene management and the implementation of good manufacturing practices (GMPs) are also critical to controlling hazardous products. Strict adherence to hygiene management and GMP requirements, including equipment cleanliness, employee hygiene, and reasonable operating procedures, can reduce sources of contamination in food products and lower the risk of harmful products. Meanwhile, proper storage and transportation of food is also critical to controlling hazardous products. Proper storage and transportation of food measures, including controlling storage temperature, humidity, and light conditions, and avoiding contamination and cross-contamination, can maintain food quality and reduce the formation of harmful products.

## 4. Detection of Harmful Products in Food Processing

Food safety is a global issue that has a significant impact on millions of people around the world every year [8]. The establishment of methods for the detection and analysis of hazardous substances in food processing is essential to provide timely and accurate analytical reports in the event of food safety accidents, and it plays an important role in the protection of public health and the prevention of diseases. Traditional methods, such as gas chromatography–mass spectrometry (GC–MS), liquid chromatography–mass spectrometry (LC–MS), and high-performance liquid chromatography (HPLC), have been developed for many years, with mature technology and wide application [9]. They can be used to analyze and detect different types of hazardous substances. These traditional methods have been validated and standardized to provide reliable and accurate analytical results and have been widely accepted by laboratories and the food industry. Compared with these traditional detection methods, nanomaterial detection methods possess the advantages of high sensitivity, high selectivity, rapid response, real-time monitoring, versatility, customizability, and improved experimental conditions [10]. These advantages make nanomaterials promising for a wide range of detection applications in various fields.

The purpose of this Special Issue was to publish high-quality articles on harmful substances in food processing, including food processing safety, generation, control, and detection of harmful substances in food processing.

## References

[1] Jennings S., Stentiford G.D., Leocadio A.M., Jeffery K.R., Metcalfe J.D., Katsiadaki I., Auchterlonie N.A., Mangi S.C., Pinnegar J.K., Ellis T. (2016). Aquatic food security: Insights into challenges and solutions from an analysis of interactions between fisheries, aquaculture, food safety, human health, fish and human welfare, economy and environment. Fish Fish..

[2] Meurillon M., Engel E. (2016). Mitigation strategies to reduce the impact of heterocyclic aromatic amines in proteinaceous foods. Trends Food Sci. Technol..

[3] Renard C.M., Watrelot A.A., Le Bourvellec C. (2017). Interactions between polyphenols and polysaccharides: Mechanisms and consequences in food processing and digestion. Trends Food Sci. Technol..

[4] Stirban A., Gawlowski T., Roden M. (2014). Vascular effects of advanced glycation endproducts: Clinical effects and molecular mechanisms. Mol. Metab..

[5] Li J., Liu D., Sun L., Lu Y., Zhang Z. (2012). Advanced glycation end products and neurodegenerative diseases: Mechanisms and perspective. J. Neurol. Sci..

[6] Lv X., Huang X., Ma B., Chen Y., Batool Z., Fu X., Jin Y. (2022). Modification methods and applications of egg protein gel properties: A review. Compr. Rev. Food Sci. Food Saf..

[7] Khan I.A., Wang C., Cai K. (2022). Hazardous substances from food processing: Formation and control, biotoxicity and mitigation. Front. Nutr..

[8] Arvanitoyannis I.S., Dionisopoulou N. (2014). Acrylamide: Formation, occurrence in food products, detection methods, and legislation. Crit. Rev. Food Sci. Nutr..

[9] Pan M., Liu K., Yang J., Hong L., Xie X., Wang S. (2020). Review of Research into the Determination of Acrylamide in Foods. Foods.

[10] Chen H., Chen X., Chen X., Lin S., Cheng J., You L., Xiong C., Cai X., Wang S. (2022). New perspectives on fabrication of peptide-based nanomaterials in food industry: A review. Trends Food Sci. Technol..

